# Supplementary material for: A method for rapid flow-cytometric isolation of endothelial nuclei and RNA from archived frozen brain tissue
Source: Lab Invest. 2021 Nov 13;102(2):204–11. doi: 10.1038/s41374-021-00698-z (PMC8784313; doi:10.1038/s41374-021-00698-z)
Supplement: Supplementary file 1 — Extended Materials and Methods [file 41374_2021_698_MOESM1_ESM.pdf]

Detailed Protocol:

Reagents

- RNase-free 10x PBS (Thermo AM9265)
- RNase inhibitor (Takara, Cat #2313A)
- RNase-free 1x PBS with RNase inhibitor
- BSA (NEB B9000S)
- Nuclei EZ lysis buffer (Sigma Nuc 101) with RNase inhibitor
- Anti-Erg 647 antibody ([EPR3864] (Alexa Fluor® 647) (ab196149))
- Anti-NeuN Cy3 antibody (Sigma MAB377C3)
- Anti-TDP43 488 antibody ([EPR5810] (Alexa Fluor® 488) (ab193842))
- DAPI (Sigma D9642-1MG)
- UltraPure Water (Invitrogen™ UltraPure™ DNase/RNase-Free Distilled Water, Cat #10977015)
- Trizol
- DNAse

Equipment

- Centrifuge with swinging bucket rotor for 15 mL and 50 mL tubes with biological safety caps for individual buckets
- Bullet Blender (Next Advance, BBY24M)
- MicroCentrifuge with biological safety cap for rotor
- BSL2 Hood
- FACS Cell Sorter
- Sterile, RNase-free Microcentrifuge Tube 1.5 mL (RINO®, Next Advance, TUBE1R5-S)
- Stainless Steel Beads 3.2 mm RNase-free (Next Advance, SSB32-RNA)
- 1.5 mL RNase-free Eppendorf tubes
- Falcon 15 mL Conical Centrifuge Tubes (Fisher Scientific, Cat #14-959-53A)
- Falcon 50 mL Conical Centrifuge Tubes (Fisher Scientific, Cat #14-432-22)
- 70 micron cell strainer
- 5 mL FACS tubes with 35 micron cell strainer caps
- Razor blades

Reagent Setup

- **RNase-free 1x PBS+RI.** Prepare a 1:10 dilution of RNase-free 10x PBS with Ultra-pure water. Add RNase inhibitor at 0.1%.
- **PBS+BSA+NaCl+RI.** Add BSA to 0.1% to RNase-free 1x PBS with 0.1% RNase inhibitor.
- **RNase-free Nuclei EZ lysis buffer+RI.** Add RNase inhibitor at 0.1% to EZ Nuclei lysis buffer
- **DAPI.** Resuspend DAPI at 1mg/mL of DAPI in UltraPure Water. Freeze in RNase-free microtube strips.
- **Antibody Dilution.** Add 100 µl PBS+BSA with RNase inhibitor in an RNase-free 1.5 mL tube. Add Anti-Erg 647 at 2.5ug/mL, Anti-NeuN Cy3 at 1:200, Anti-TDP43 488 at 2.5ug/mL, and DAPI at 1:2000

Equipment setup

Cool Reagents
- Place all reagents on ice or store in 4℃.

RINO Tubes
- Add eight 3.2 mm stainless steel beads to each 1.5 mL RINO tube. Then add 700 µL of EZ Nuclei Lysis buffer with RNase inhibitor and keep on ice.

Bullet Blender
- Keep Bullet Blender in a cold room (4℃).

Centrifuges
- Bring all centrifuges to 4℃.

70 Micron Cell Strainer
- Remove cap from 50 mL tube and place on a 70 micron cell strainer. Keep on ice

35 Micron Cell Strainer
- Place 5 mL tubes with 35 micron cell strainers on ice.

Methods:

***Critical:** All steps involving human brain tissue are to be done in a BSL2 hood. All steps should be done as quickly as possible, so equipment, tubes, and reagents should be prepped and cooled in advance, when possible.

Homogenization of brain tissue
- Thaw tissues on ice, and place ~200 mg brain tissue (half mouse brain) in 1.5 mL RINO tube with 700 µl EZ Nuclei Lysis Buffer with RNase inhibitor and eight 3.2 mm stainless steel beads. Allow thaw on ice.
- Place RINO tube in Bullet Blender and homogenize at setting 4 for 4 minutes at 4℃.

Cell lysis and washing of nuclei
- Move liquid (avoiding beads) to 15 mL tube. Repeatedly wash the RINO tube with EZ Nuclei Lysis Buffer (once with 300 µl and 4 times with 1 mL), transferring the liquid to the 15 mL tube each time.
- Mix sample and incubate 2 minutes on ice. Centrifuge at 700 *g* for 5 minutes at 4℃.
- <u>Important</u>: When using human brain tissue, securely cover the sample inside the centrifuge. Transfer the sample to and from the centrifuge and the chemical fume hood with the cover on.
- Remove supernatant and resuspend in 5 mL EZ Nuclei Lysis Buffer. Mix and incubate 2 minutes on ice. Centrifuge at 700 *g* for 5 minutes at 4℃.
- Remove supernatant and bring pellet up to 300uL in PBS and move to an RNase-free 2mL tube.
- Add 300 µL 1% PFA in PBS (approximately equal parts sample and 1% PFA, resulting in a 0.5% concentration). Incubate exactly 1 min then add 1 mL PBS+RNase inhibitor and spin at 700 *g* 3 min at 4°C.
- Remove supe and resuspend in 1mL PBS+BSA+RNase inhibitor+500mM NaCl. Spin at 700 *g* 5 min at 4°C

Filtering nuclei
- Remove supernatant and resuspend in 1 mL PBS+BSA+RI+NaCl.

- Filter sample through 70 micron filter into 50 mL tube, ensuring that as much of the sample passes through as possible by moving to a new spot in the filter each time it clogs.
- Filter flow-through through 35 micron filter.

Nuclei staining

- Move the flow-through to a new RNase-free 1.5 mL tube. Separate 1/20 of the flow-through on ice for an unstained control and single antibody controls. Centrifuge all at 700 *g* for 5 min at 4℃.
- Remove supernatant and resuspend in staining buffer, or staining buffer with one or more antibodies (e.g., 100 µl of PBS+BSA+RNase inhibitor+500 mM NaCl with Anti-Erg 647 (1:200), Anti-NeuN Cy3 (1:200), Anti-TDP43 488 (1:200), and DAPI (1:2000)). Incubate on ice for 15 min.

Washing and preparing nuclei for FACS sorting

- Add 1 mL of PBS+BSA+RNase inhibitor+NaCl. Centrifuge at 700 *g* for 5 min at 4℃.
- Remove supernatant and resuspend in 500 µl PBS+BSA+RI+NaCl. Add 300 U of DNAse, incubate for 5 min on ice.
- Filter through 35 micron filter into a 5 mL FACS tube.
- Sort Erg-positive cells and NeuN-positive nuclei from DAPI (single nuclei). Collect NeuN+, and Erg+ nuclei, spin down at max, remove supe and store pellet at -80°C.
- Retrieve RNA using RNeasy FFPE Kit (Qiagen 73504).
