# Supplementary material for: A method for rapid flow-cytometric isolation of endothelial nuclei and RNA from archived frozen brain tissue
Source: Lab Invest. 2021 Nov 13;102(2):204–11. doi: 10.1038/s41374-021-00698-z (PMC8784313; doi:10.1038/s41374-021-00698-z)
Supplement: Supplementary file 2 — SI Figures [file 41374_2021_698_MOESM2_ESM.pdf]

Group	***Sex***	***Age***	***PMI***
Young	M	19	13
Young	M	20	18
Young	M	22	13
Old	M	68	28
Old	M	69	23
Old	M	71	18
Dementia	M	58	3
Dementia	M	65	12
Dementia	M	67	16
Dementia	M	72	5
Dementia	M	74	14
Old-test	F	87	4

SI Table 2. Archived human brain tissue from NIH NeuroBioBank
Frozen cortical tissues (Brodmann Area 10) were obtained from each of the indicated samples. Age and sex are indicated, as well as post-mortem interval (PMI). Tissues were cut into 200mg pieces, and separately analyzed by staining and flow-cytometry using a protocol with and without brief 4% PFA fixation.

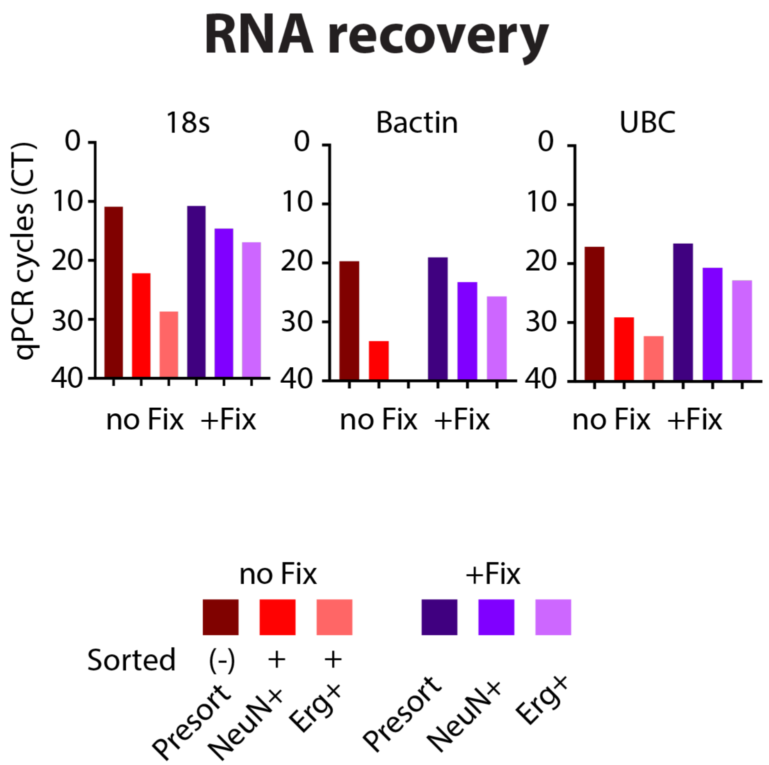

Supplemental Figure 1. Fixation of RNA in nuclei allows improved recovery after sorting. Recovery of RNA from unsorted nuclei from mouse brain (200mg tissue), and from nuclei after sorting, with or without fixation. 10% of the nuclei from the tissues were used to prepare cDNA for the unsorted fraction and all of the nuclei were used to prepare cDNA for the sorted fractions. Graphs show the threshold cycle (CT) from quantitative PCR analysis of the indicated genes.

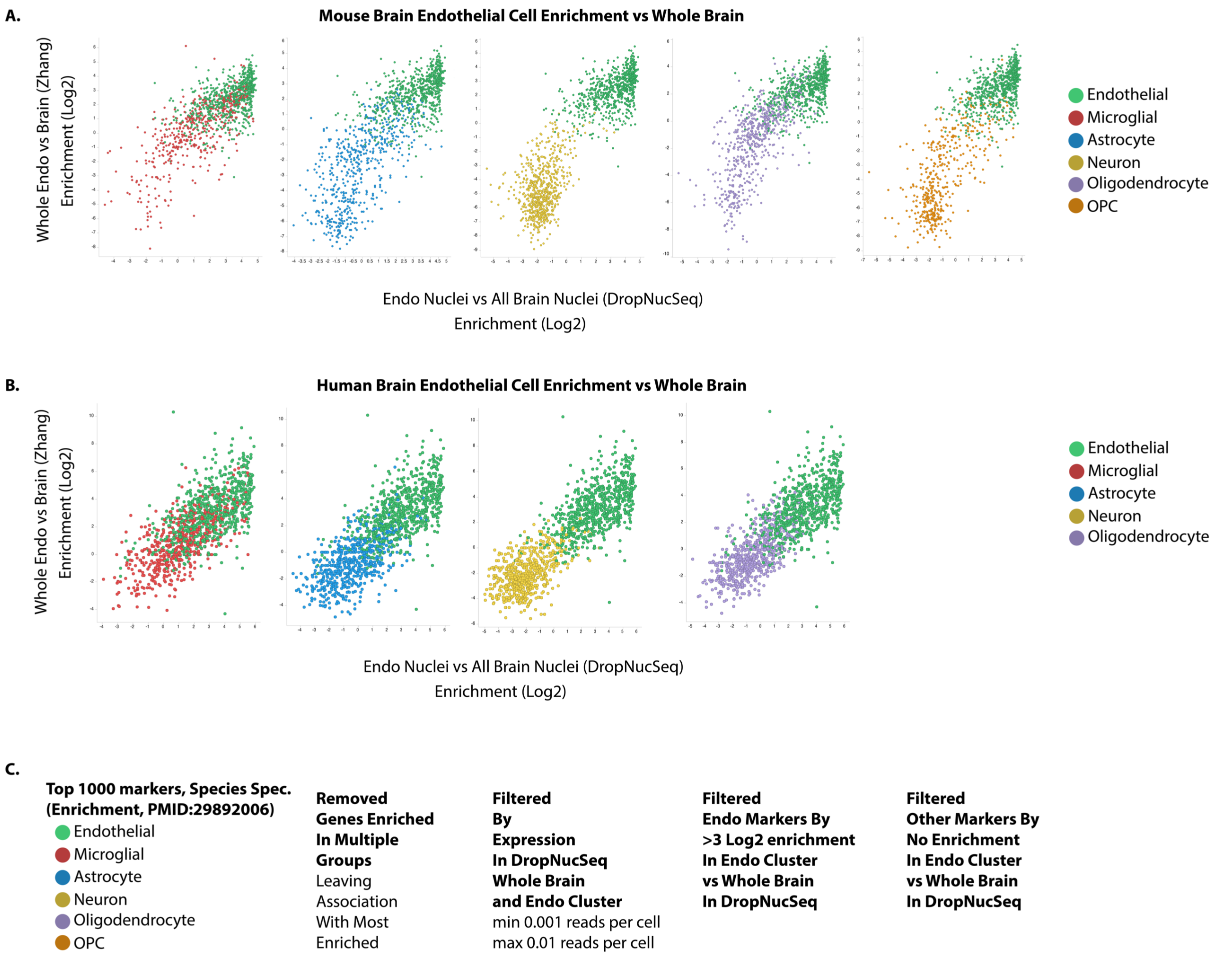

Supplemental Figure 2. Cell markers used in enrichment analysis.
Plots show the endothelial enrichment of individual genes (points on the plot), for genes previously identified as enriched in the indicated cell types from a meta-analysis of cell-specific RNA sequencing experiments in (A) mouse and (B) human brain tissues. Datasets used to plot enrichment were (A) Zhang whole endothelial cell isolation vs. whole mouse cortex (PMID: 25186741) and DropNucSeq data from endothelial cluster 21 vs. all nuclei (all clusters) (PMID: 28846088) and (B) Zhang whole endothelial cell isolation vs. bulk human cortex (PMID: 26687838) and DropNucSeq data from endothelial cluster 16 vs. all nuclei (all clusters) (PMID: 28846088). (C) The marker panel was trimmed as described for mouse and human markers individually, resulting in the pared list of genes used as markers for enrichment analysis.

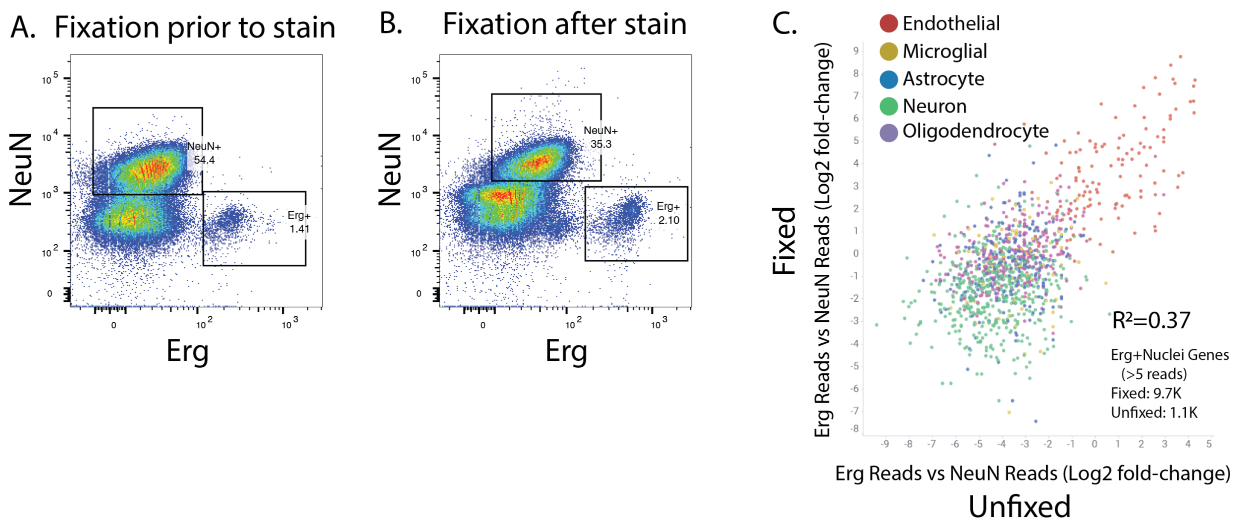

Supplemental Figure 3. Comparison of fixed and unfixed samples.

Flow cytometry plots showing result from staining samples before (A) or after to fixation (B). Analysis of RNA-seq data from fixed or unfixed mouse Erg+ nuclei (as shown in Figure 2). Unfixed RNA was prepared by polyA preparation (SMART-Seq), while fixed RNA was prepared by random priming (SMARTer Total RNA) and filtered for junction spanning reads before quantitation (as described in methods). Enrichment of transcripts relative to NeuN nuclei was performed, based on the relative proportion of gene transcripts per million from each sample type, and presented as the log 2 transformed fold-change. Inset shows the total number of genes from each Erg+ sample type with >5 reads per gene.

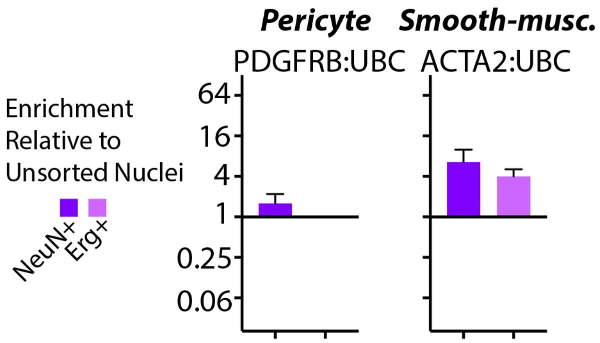

Supplemental Figure 4. Mural cell markers are not enriched in the Erg+ fraction.
Enrichment of cell type-specific RNA from the sorted Erg+ or NeuN+ nuclei versus unsorted nuclei by quantitative PCR. The fold-change increase in each transcript, relative to unsorted nuclei is shown.

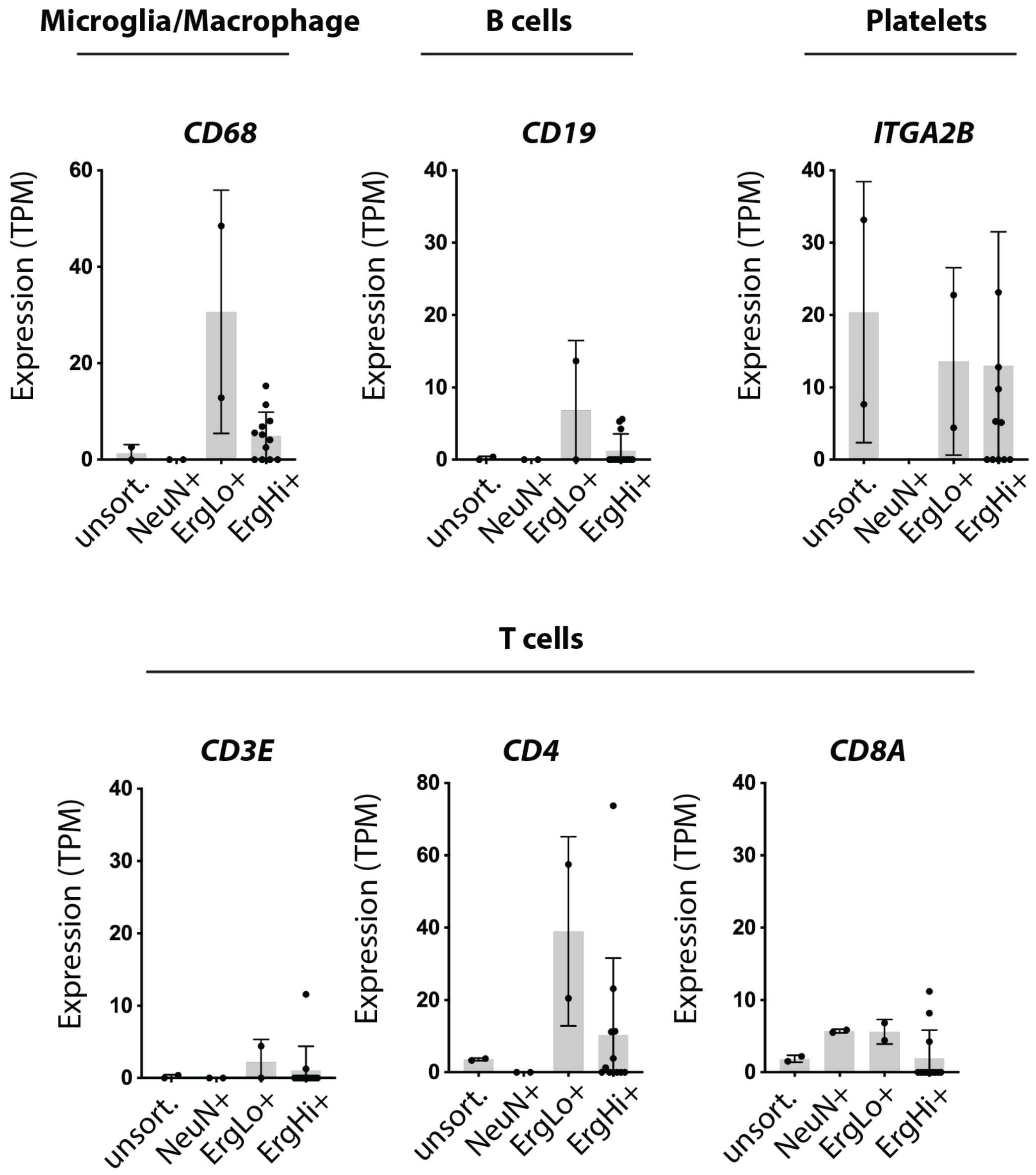

Supplemental Figure 5. Expression of canonical hematopoietic markers in different sorted populations.

Graphs show the expression levels of the indicated genes in sorted populations of nuclei (NeuN+, ErgLo+ or ErgHi+) or unsorted nuclei (unsort.) from human brain tissue. As described in methods, read analysis was first filtered for junction spanning reads.

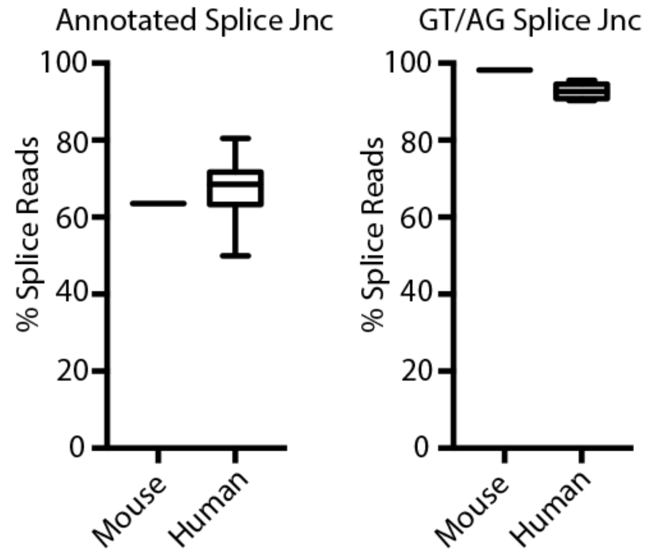

Supplemental Figure 6. Annotation of splice junction reads from Erg+ samples.
Graphs show the STAR analysis of splice junction reads, and the percentage mapping to known splice junctions in the Hg38 annotation file, and those with GT/AG junction (canonical splice junction dinucleotides).

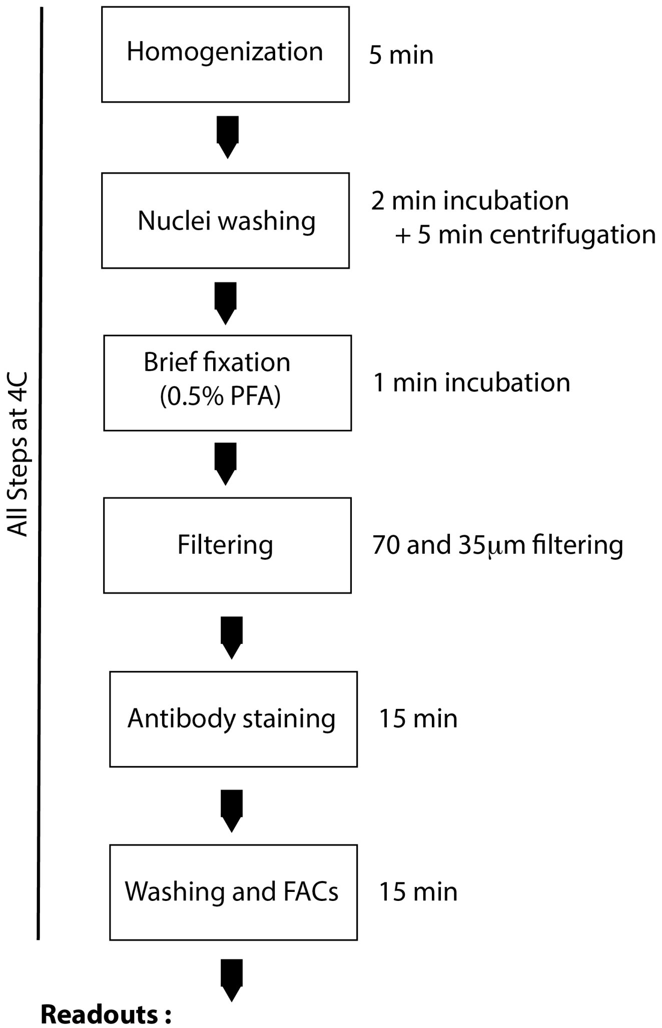

Readouts :

Flow cytometry separation of
- Erg+ (0.5-3% DAPI+)
- NeuN+ (20-50% DAPI+)

Random primed qPCR of extracted RNA
- 10-20 fold enrichment of CD31, CDH5

Supplemental Figure 7. Outline of the protocol to accompany extended methods
